# Expression of CD39 Is Correlated With HIV DNA Levels in Naïve Tregs in Chronically Infected ART Naïve Patients

**DOI:** 10.3389/fimmu.2019.02465

**Published:** 2019-10-17

**Authors:** Jin-Wen Song, Hui-Huang Huang, Chao Zhang, Hong-Ge Yang, Ji-Yuan Zhang, Ruo-Nan Xu, Lei Jin, Ming Shi, Fu-Sheng Wang, Yan-Mei Jiao

**Affiliations:** ^1^Treatment and Research Center for Infectious Diseases, The Fifth Medical Center of Chinese PLA General Hospital, Beijing, China; ^2^Savaid Medical School, University of Chinese Academy of Sciences, Beijing, China

**Keywords:** HIV DNA, CD39, nTreg, mTreg, disease progression

## Abstract

**Background:** Treg cells represent important viral reservoirs during chronic HIV infection. CD39 is closely involved in Treg-mediated immunosuppressive effects. However, CD39 expression on nTregs and mTregs and a relationship with HIV DNA levels during HIV infection is still unclear. In this study, we analyzed the distribution of HIV DNA in Treg subsets and the association between HIV DNA and CD39 expression on Treg subsets.

**Methods:** Sixty-two HIV-infected patients with different HIV stages and 14 uninfected individuals were enrolled. nTregs (CD4^+^CD25^+^CD127^low^CD45RO^−^) and mTregs (CD4^+^CD25^+^CD127^low^CD45RO^+^) were isolated by magnetic selection and flow cytometric sorting. HIV DNA was quantified by real-time polymerase chain reaction (PCR). CD39 expression on nTregs and mTregs was analyzed by flow cytometry.

**Results:** Higher levels of HIV DNA were detected in mTregs than those in nTregs during chronic HIV infection. The frequency of CD39^+^ nTregs and HIV DNA levels in nTregs were increased in patients with advanced HIV infection. Furthermore, HIV DNA levels in nTregs correlated positively with CD39^+^ nTreg frequency. CD39^+^ nTreg frequency was also increased in immune non-responders.

**Conclusions:** mTregs and nTregs are both important reservoirs of virus during chronic HIV infection and HIV DNA levels increase in nTregs in patients with advanced HIV infection. We observed increased frequency of CD39^+^ nTregs and HIV DNA levels in nTregs in patients with advanced HIV infection. HIV DNA levels in nTregs correlated positively with CD39^+^ nTreg frequency.

## Introduction

Despite the success of anti-retroviral therapy (ART) in suppressing human immunodeficiency virus (HIV) replication to undetectable levels in plasma, complete eradication of HIV is currently not achievable (1). The main obstacle to a cure for patients with HIV infection is viral persistence in some long-lived infected cells, which form a reservoir of virus (2). The HIV reservoir persists despite ART and viremia rebounds rapidly when ART is interrupted (3).

HIV DNA levels indicate the size of the viral reservoir (4). It has been reported that central memory and transitional memory CD4^+^ T cells are the major reservoir for HIV (5). However, to a lesser extent, naïve CD4^+^ T cells are also found to contain integrated HIV DNA (6). Thus, considering the longevity and stable HIV DNA levels of naïve CD4^+^ T cells, they may also represent an important part of the HIV reservoir (6).

Regulatory CD4^+^ T cells (Tregs) are commonly characterized by high expression of CD25 (IL-2Rα) and a lack of CD127 (IL-7α) expression on the cell surface, and intracellular expression of the transcription factor Foxp3 (7–9). During HIV infection, Tregs are preferentially preserved compared to conventional CD4^+^ T cells (10–12). And, Treg cells have plasticity, it might become unstable under certain inflammatory conditions and might adopt a phenotype that is more characteristic of effector CD4^+^ T cells (13). Thus, the role of Tregs in HIV pathogenesis is complicated. On one hand, Tregs may suppress anti-HIV specific CD4^+^ and CD8^+^ T cell responses by inhibiting cell proliferation and effector molecule production (12, 14, 15). However, on the other hand, Tregs can suppress HIV-induced chronic inflammation and inhibit HIV infection and replication (12, 16). Moreover, Tregs are also targets of HIV infection (17) and serve as a viral reservoir (18, 19). In a simian immunodeficiency virus (SIV)-infected rhesus macaque model, higher levels of SIV DNA were detected in the mucosal Treg subset than those in non-Treg CD4^+^ cells (20). In HIV-infected patients, we also showed that HIV DNA levels in Tregs were approximately 10-fold higher than those in non-Tregs CD4^+^ cells (19). Furthermore, replication competent virus has been reactivated from Tregs isolated from HIV-infected individuals on long-term ART (17, 19). Moreover, HIV-specific CD134^+^CD39^+^ Treg correlated positively with viral load, which further suggest that Treg might represent a potent HIV-1 reservoir (21). Thus, more research is needed to investigate HIV persistence in naïve CD4^+^CD25^+^CD127^low^CD45RO^−^ Tregs (nTregs) and their memory counterparts, CD4^+^CD25^+^CD127^low^CD45RO^+^ Tregs (mTregs) during the course of the infection.

CD39, a member of the ectonucleoside triphosphate diphosphohydrolase (E-NTPDase) family, converts ATP and ADP to AMP (22), which is then hydrolyzed to adenosine by CD73. The accumulated extracellular adenosine can signal through binding with one of four adenosine receptors (A1R, A2AR, A2BR, and A3R) (23). The binding of adenosine with A2AR and A2BR can exert immunosuppressive function by inducing intracellular AMP (cAMP) signaling (24). CD39 can act in concert with CD73 to suppress the extracellular ATP-mediated proinflammatory effect. It has been reported that CD39 expression is significantly higher in Tregs than in non-Treg CD4^+^ T cells and CD39 is involved in Treg-mediated suppression of HIV infection (16). In addition, CD39 expression on Tregs and adenosine levels in a natural SIV host were found to be higher than those in non-natural host monkeys, in addition, immune activation and inflammation markers inversely correlated with adenosine, thus CD39 expression on Tregs might play a critical role of in suppressing immune activation and inflammation (25). Furthermore, Treg cells can regulate IL-2 expression via CD39/adenosine pathway in HIV infection (26). Collectively, these lines of evidence suggest that CD39 is closely involved in Treg-mediated immunosuppressive effects. However, the correlations of CD39 expression on nTreg and mTregs with HIV DNA levels during HIV infection is still unclear.

In this study, we evaluated HIV DNA levels and CD39 expression on nTregs and mTregs in HIV-infected individuals at different stages of infection. We found that HIV DNA levels in nTregs and CD39 expression on nTregs from patients with advanced HIV infection were significantly increased and CD39^+^ nTreg frequency correlated positively with HIV DNA levels in nTregs.

## Materials and Methods

### Study Population and Samples

A total of 69 HIV-infected patients and 14 HIV-uninfected healthy individuals were recruited at Beijing 302 Hospital, China. Peripheral blood mononuclear cells (PBMCs) were prepared from EDTA anti-coagulated venous blood by Ficoll-Hypaque (MD Pacific Biotechnology, Tianjin, China) density gradient centrifugation. All blood samples were collected with the approval of the Beijing 302 Hospital Research Ethnics Committee. Study subjects gave written informed consent to participate in accordance with the Declaration of Helsinki.

The HIV-infected patients was divided into the subgroups described in Table 1 as follows: (1) Treatment naïve (*n* = 35): without ART; (2) Complete responders (CRs) (*n* = 20): received ART for more than 2 years with peripheral CD4^+^ T cell counts above 350 cells/μL and plasma HIV-1 RNA <80 copies/mL; (3) Immune non-responders (INRs) (*n* = 14): received ART for more than 2 years with peripheral CD4^+^ T cell counts below 200 cells/μL and plasma HIV-1 RNA <80 copies/mL. The ART regimen included two nucleoside reverse transcriptase inhibitors (NRTIs) plus one non-nucleoside reverse transcriptase inhibitor (NNRTI). Exclusion criteria included pregnancy, hepatitis B virus, hepatitis C virus, tuberculosis, or Dengue virus infections, and moribund status.

**Table 1:** Characteristics of patients in this study.

**Patients**	**Healthy controls**	**Treatment naive**			**Complete responders to ART(CRs)**	**Immune non-responders to ART(INRs)**
		**CD4 >500**	**200 < CD4 ≤500**	**CD4 ≤200**		
Cases (*n*)	14	12	16	7	20	14
Age (years)	38 (24–60)	38 (24–51)	40 (22–55)	43 (33–54)	38 (25–59)	41 (27–58)
Gender (male/female)	10/4	8/4	13/3	5/2	15/5	10/4
CD4 count (cells/μL)	NA	679 (542–950)	407 (214–484)	117 (54–184)	543 (472–698)	127 (89–198)
HIV load (copies/mL)	NA	51,504 (3,900–337,236)	114,202 (2,090–380,461)	89,153 (1,530–349,000)	<500	<500

*Data are expressed as the median (range), unless otherwise stated. ART, highly active antiretroviral therapy; HIV, human immunodeficiency virus; NA, not applicable*.

The HIV-1 RNA levels in plasma were quantified using HIV-1 RT-PCR Assay V2 (Qiagen, Hilden, Germany) according to the manufacturer's instructions. PCRs were performed using a CFX96 real-time polymerase chain reaction (PCR) system (Bio-Rad, Hercules, CA, USA).

### Flow Cytometric Characterization of T Cells

PBMCs were stained with the Acqua Live/Dead Stain (Invitrogen, Carlsbad, CA, USA) for 20 min at room temperature. Then, the following panel of anti-human fluorochrome-conjugated antibodies were used for flow cytometric characterization of T cells: anti-CD3-PerCP (BD Biosciences, San Diego, California, USA), anti-CD4-APC (BD Biosciences), anti-CD4-PE-cy7 (BD Biosciences), anti-CD25-BV421 (BD Biosciences), anti-CD127-FITC (eBioscience, Waltham, MA, USA), anti-CD45RO-BV510 (eBioscience), CD39-PE (eBioscience). After incubation for 30 min at 4°C in the dark, the cells were washed with FACS buffer and fixed. For intracellular staining, cells were fixed and permeabilized using Foxp3 staining kit (eBioscience) then stained with anti-Foxp3-APC (BD Biosciences). Data were acquired on a BD-FACSCanto (BD Biosciences) and analyzed using FlowJo software V10 (Tree star lnc., Ashland, OR, USA).

### Cell Sorting

CD4^+^ T cells were isolated from cryopreserved PBMC of HIV-infected patients using a magnetic negative selection kit (Stem Cell Technologies, Vancouver, Canada) following manufacturer's protocol. Purified CD4^+^ T cells were stained with antibodies against CD25, CD127 and CD45RA. After 30 min incubation at 4°C in the dark, cells were washed and sorted using the BD-FACSAria II (BD Biosciences).

### HIV DNA Quantification

HIV DNA was extracted using Qiagen QIAsymphony DNA Mini kit (Qiagen) according to manufacturer's instructions. Quantification of HIV DNA was performed by quantitative real-time PCR using a SUPBIO HIV Quantification kit (SUPBIO, Guangzhou, China) (27), with a quantification range of 5 – 10 × 10^6^ copies per 10^6^ PBMC.

### Statistical Analysis

Statistical analysis was carried out using GraphPad Prism 7.0 (GraphPad Software, San Diego, CA, USA). Comparisons between two groups were performed using the Mann–Whitney *U*-test. A non-parametric Kruskal–Wallis test was used to test for differences between more than two groups. Correlations were determined using the Spearman rank correlation test. All data were expressed as mean ± standard error of the mean (SEM). *P* < 0.05 were considered to indicate statistical significance.

## Results

### CD39^+^ nTregs Frequency Is Increased in Patients With Advanced Stage HIV Infection

**Figure 1:**
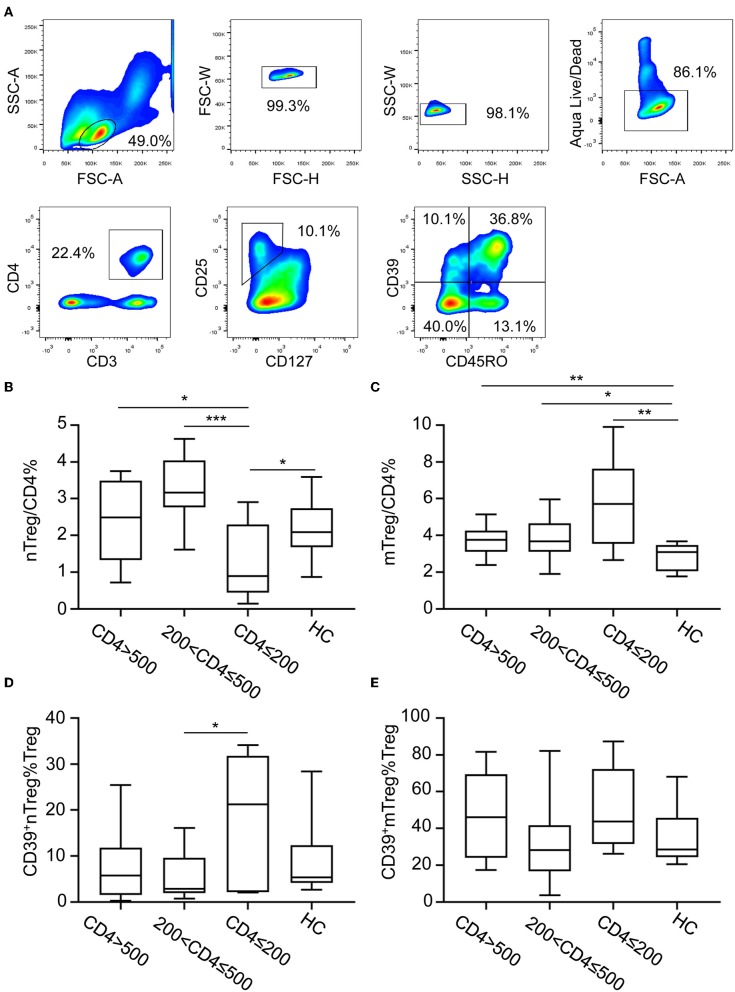
CD39 expression on nTreg and mTreg cells of HIV-infected participants. **(A)** Gating strategy for flow cytometry analysis. Cells were first gated for lymphocyte and singlets. Live cells were discriminated using Aqua Live/Dead staining kit. Tregs cells were further identified base on CD25 and CD127 expression and CD39 expression on nTreg and mTreg cells were analyzed. **(B–E)** Anti-retroviral therapy naïve HIV-infected patients were stratified into three groups according to absolute CD4^+^ T cell counts: CD4 ≤200 cells/μL, 200< CD4 ≤500 cells/μL, and CD4 >500 cells/μL. The frequency of nTreg **(B)** and mTreg **(C)** cells among total CD4^+^ T cells in each group. Frequencies of CD39^+^ nTregs **(D)** and CD39^+^ mTregs **(E)** among Treg cells in each group. Statistical significance between two groups was determined by a Mann–Whitney *U*-test. *P < 0.05, **P < 0.01, ***P <0.001.

CD127 and CD25 expression was used to discriminate Tregs from other CD4^+^ T cells (Figure 1A). Two subsets of Tregs were identified based on the expression of CD45RO: nTregs (CD127^−^CD25^+^CD45RO^−^) and mTregs (CD127^−^CD25^+^CD45RO^+^) (Figure 1A). Both nTregs and mTregs express high levels of Foxp3 (Supplementary Figure 1). ART-naïve HIV-infected participants were grouped into three categories according to their CD4^+^ T cell counts (CD4^+^ T ≤200 cells/μL, *n* = 7; 200 < CD4^+^ T ≤500 cells/μL, *n* = 16; CD4^+^ T >500 cells/μL, *n* = 12) (Table 1). The frequencies of nTregs and mTregs in different groups of HIV-infected patients are shown in Figures 1B,C, respectively. The frequency of nTregs in advanced stage patients with CD4^+^ T counts <200 cells/μL was decreased compared with those detected in patients in the other stages of infection, as well as in healthy uninfected individuals. In contrast, the frequency of mTregs increased in all stages of HIV infection, especially in the advanced stage, compared with healthy uninfected controls. CD39 expression is preferentially expressed on Tregs; therefore, we further analyzed CD39 expression on nTregs and mTregs by flow cytometry (Figures 1D,E). In accordance with previous reports (28), we also observed that CD39 was preferentially expressed on memory Tregs. Interestingly, we found that the frequency of CD39^+^ nTregs among Tregs was significantly increased in advanced stage HIV infection. However, there were no significant differences in the frequency of CD39^+^mTregs among Tregs, irrespective of the HIV infection stage.

### HIV DNA Levels Are Increased in nTregs in Advanced Stage HIV Infection

**Figure 2:**
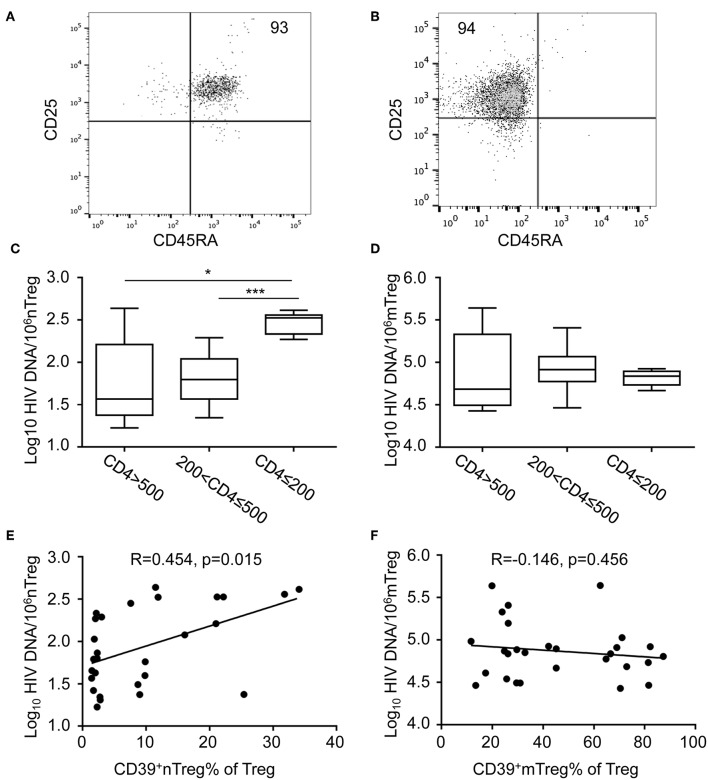
The quantification of HIV DNA within sorted nTreg and mTreg subsets. **(A,B)** Flow cytometric analysis of CD45RA and CD25 expression on purified nTreg **(A)** and mTreg **(B)** cells of a representative HIV-infected individual. Numbers indicate the percentage of gated cells. **(C,D)** Real-time PCR quantification of HIV DNA levels in nTreg **(C)** and mTreg **(D)** cells. HIV DNA levels (copies/10^6^ cells) in each group are represented on a log_10_ scale. **(E,F)** The relationship between the frequencies of **(E)** CD39^+^ nTregs and **(F)** CD39^+^ mTregs with HIV DNA levels in nTreg and mTreg cells, respectively. Associations were evaluated using Spearman correlation tests. *P* and Spearman's rho values are presented. The fitted line superimposed on the relevant graph, was estimated through linear regression. *P < 0.05, ***P < 0.001.

HIV DNA is a marker of HIV persistence and is predictive of clinical progression in the absence of ART (29, 30); however, changes in HIV DNA expression levels in nTregs and mTregs at different stages of HIV infection remain poorly understood. To investigate this issue, we sorted nTregs and mTregs from PBMC of HIV-infected patients according to their expression of CD25, CD127, and CD45RA. The purity of nTregs and mTregs was confirmed to exceed 90% by flow cytometry (Figures 2A,B) and HIV DNA levels were determined by qRT-PCR. The distribution of HIV DNA in mTregs was much higher than that in nTregs (Figures 2C,D). Surprisingly, HIV DNA levels in nTregs in patients with advanced stage HIV infection were significantly increased compared with the levels detected in the other groups (Figure 2C). However, there were no significant differences in HIV DNA levels in mTregs from the different groups of HIV-infected patients (Figure 2D).

### Relationship Between HIV DNA Levels in nTregs and CD39^+^ nTreg Frequency

The frequency of CD39^+^ nTregs and HIV DNA levels in nTregs increased during the advanced stage of HIV infection. Therefore, we analyzed the relationship between HIV DNA levels and CD39 expression in Treg subsets. Of note, we observed a significant positive correlation between the frequency of CD39^+^ nTregs and HIV DNA in nTregs (*r* = 0.454, *P* = 0.015, Figure 2E). However, there was no significant correlation between the frequency of CD39^+^ mTregs and HIV DNA levels in mTregs (*r* = −0.146, *P* = 0.456, Figure 2F). As expected, the counts of both CD39^+^ nTregs and CD39^+^ mTregs correlated positively with current CD4^+^ T cell counts (*r* = 0.4046, *P* = 0.0176; *r* = 0.6729, *P* < 0.001, Supplementary Figures 2C,D, respectively). However, there were no significant correlations between CD4^+^ T cell counts and the frequencies of CD39^+^ nTregs or CD39^+^ mTregs (Supplementary Figures 2A,B). In addition, we observed significant correlations of viral load with the frequency of CD39^+^ nTregs, but not with CD39^+^ mTregs (Supplementary Figures 2E,F, respectively).

### CD39^+^ nTreg Frequency Is Increased in INRs

**Figure 3:**
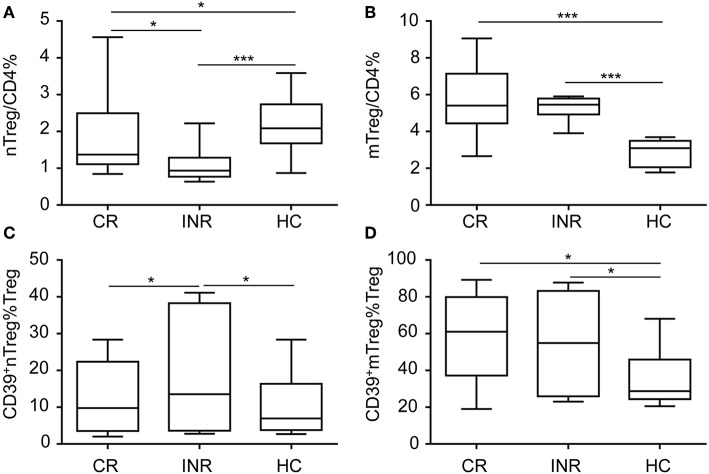
CD39^+^ nTreg frequency is increased in immune non-responder (INR) individuals. Comparisons of **(A)** nTreg and **(B)** mTreg frequencies among complete responders (CRs), INRs and HIV-uninfected controls. Comparisons of **(C)** CD39^+^ nTreg frequencies and **(D)** CD39^+^ mTreg frequencies among CRs, INRs and HIV-uninfected controls. *P < 0.05, ***P < 0.001.

To further determine CD39 expression on nTregs and mTregs in HIV-infected subjects with different degrees of immune restoration after long-term ART, we recruited 20 CRs and 14 INRs treated with ART for more than 2 years. Compared with healthy uninfected controls, the nTreg frequency was decreased in both CRs and INRs (Figure 3A). Furthermore, nTreg frequency was lower in INRs compared with that in CRs. In contrast, mTreg frequency was increased in both CRs and INRs (Figure 3B). Of particular interest, CD39^+^ nTregs frequency was increased in INRs compared with that in CRs and uninfected healthy controls (Figure 3C). However, there was no significant difference in CD39^+^ mTreg frequency between INRs and CRs (Figure 3D). These data suggest that long-term ART did not normalize the frequencies of CD39^+^ nTregs and CD39^+^ mTregs, which may play a role in immune failure.

## Discussion

In this study, we demonstrated that the frequency of nTregs is decreased in patients with advanced stage HIV infection and INRs. We also showed the extensive distribution of HIV DNA in both nTregs and mTregs. Of note, HIV DNA levels were significantly increased in nTregs in patients with advanced stage HIV infection. As CD39 is a functional marker of Tregs (31), we further investigated the relationship between CD39 expression on Treg subsets and HIV DNA levels. We found that the frequency of CD39^+^ nTregs correlated positively with HIV DNA levels, and the frequency of CD39^+^ nTregs was significantly increased in patients with advanced HIV infection and INRs.

Our results further confirmed that Tregs constitute a potent reservoir of virus and contribute to HIV persistence in CD4^+^ T cells (19). Recently, McGary et al. reported that CTLA-4^+^PD-1^−^ memory CD4^+^ T cells contribute critically to viral persistence in ART-suppressed SIV-infected rhesus macaque monkeys, while CTLA-4 is also a marker of Tregs (32). Anti-CTLA-4 antibodies have been shown to deplete Tregs (33). Notably, a transient decrease in HIV RNA was observed in an HIV-infected patient being treated for melanoma after infusion of an anti-CTLA-4 antibody, ipilimumab (34). Studies have shown that the distribution of latent HIV levels are approximately 10-fold higher in memory T cells compared with those in naïve T cells (35–37). In this study, we analyzed the distribution of HIV DNA in two subsets of Tregs from ART-naïve individuals stratified by CD4^+^ T cell counts and demonstrated that the distribution of HIV DNA is approximately 1,000-fold higher in mTregs than in nTregs (Figure 2). The ongoing HIV replication in ART-naïve patients and the susceptibility of memory CD4^+^ T cells to HIV infection may contribute together to the high HIV DNA levels in mTregs. Even though the absolute number of infected nTregs is significantly smaller than that of infected mTregs, nTregs showed the capacity to expand significantly following stimulation and therefore, may represent an important reservoir of virus. In addition, we observed that HIV DNA levels in nTregs were significantly increased in patients with advanced infection (Figure 2C). However, the size of the pool of cell carrying replication-competent HIV virus might be overestimated by determining total HIV DNA, as a result of defective HIV virus (38). The destruction of nTregs in advanced HIV-infected patients might further increase immune activation and promote disease progression. Interestingly, we also observed that the frequency of CD39^+^ nTregs correlated positively with HIV DNA levels in this population (Figure 2E), although the underlying mechanism remains to be clarified. Tregs suppress the activation of HIV-infected CD4^+^ T cells which reversing these T cells into resting T cells (39). In addition, Tregs can suppress the HIV-specific CD8^+^ T cells, which further contributing to HIV reservoir formation. Importantly, the suppressive function of Tregs is mediated by the expression of surface proteins, primarily CTLA-1 and CD39. Thus, CD39^+^nTregs might play a role in promoting HIV reservoir formation in naïve CD4^+^ T cells. What's more, as reported by Zerbato et al., although the frequency of HIV infection is low in naive CD4^+^ T cells, they still harbor a large inducible reservoir which is as much to memory CD4^+^ T cells (40).These results suggest that future strategies targeting the HIV reservoir should also target nTregs. And, further research is warranted to investigate the effect of manipulation of CD39 on HIV latency and HIV disease progression.

Extracellular ATP acts as danger-associated molecular pattern (DAMP) and binds to purinergic receptors to trigger signaling cascades that induce an inflammatory response (23). CD39 is the rate-limiting enzyme in the ATP/ADP-adenosine pathway (41). CD39 combined with CD73 function to induce a shift from an ATP-mediated proinflammatory environment to an immunosuppressive milieu stimulated by adenosine (22). In addition, CD39 is involved in the regulatory effect of Tregs (42). In contrast to murine CD39^+^ Tregs, surface co-expression of CD73 on human circulating CD39^+^ Tregs is rare (43). CD39^+^ Tregs are required to interact with CD73-expressing immune cells to hydrolyze ATP to immunosuppressive adenosine. Yan Tang et al. showed that CD39 was expressed preferentially on memory Tregs (28). In accordance with this report, we also showed higher CD39 expression on mTregs during HIV infection (Figure 1A). In non-human primate, the CD39 and CD73 coexpression on Tregs were higher in intestine than in lymph node or blood. Besides, the baseline level of CD39 and CD73 co-expression on Treg cells were higher than non-natural host monkeys. Upon SIV infection, a significant increase in the levels of CD39 and CD73 coexpresssion on Tregs in the intestine were observed in non-natural host monkey (25). In addition, higher CD39^+^ nTregs frequencies were observed in patients with advanced stage HIV infection and INRs (Figures 1D, 3C). The expansion of CD39^+^ Tregs correlates with lower CD4^+^ T cell counts in HIV-infected patients (44); therefore, it can be speculated that the increased frequencies of CD39^+^ Tregs may be attributed, at least partially, to the increase in CD39^+^ nTregs.

HIV infection is characterized by CD4^+^ T cell depletion and chronic inflammation (45–47). Interestingly, recent studies showed that CD39^hi^ Tregs sustained their suppressive ability and exhibited high Foxp3 expression in an inflammatory environment (48). The proportion of CD39^+^ Tregs is significantly increased during HIV infection in viremic patients, ART-treated individuals and long-term non-progressors (LNTP) (44, 49). However, elite controllers present proportions of CD39^+^ Tregs similar to those of healthy donors (49). In addition, CD39 expression on CD4^+^ T cells is highly variable between individuals and rs11188513-C SNP of CD39 was negatively correlated with HIV-1 disease progression (44). Furthermore, Th17/Treg ratio dysbalance is involved in loss of epithelial barrier integrity and HIV disease progression (11). The expression of CD39 on a subset of human Tregs might be able to effectively self-control the induction of Th17 cells *per se* (50). The increased CD39^+^ Treg frequency might be an important mechanism by which Tregs execute their immunosuppressive function in a chronic inflammatory environment during HIV infection.

We acknowledge several limitations to our study. First, these experiments are performed in peripheral blood not tissues. Also, cell markers to identify Treg cells are limited. Foxp3 can also be included to identify Treg cells, but we could not isolate Treg cells based on Foxp3 to determine HIV DNA in Treg cells. And we did not detect IL10 secretion in Treg cells as well as in plasma. In addition, we detected total DNA, future studies will also be need to determine integrated, non-integrated DNA and replication-competent HIV. Moreover, we did not measure the levels of HIV-DNA in sorted CD39^+^ Tregs, but we tried the HIV-flow assay to detect p24 levels in CD39^+^ Tregs, however, we did not succeed in depicting p24 positive cells in the CD39^+^ gate (data not shown), future studies might be needed to determine the HIV-DNA levels in CD39^+^ Tregs.

In this study, we showed that both mTregs and nTregs act as HIV reservoirs during chronic HIV infection. Both HIV DNA levels and CD39 expression in nTregs increased in patients with advanced stage infection. Furthermore, we identified a positive correlation between CD39 expression and HIV DNA levels in nTregs. Considering the longevity and plasticity of nTregs, more detailed studies should focus on the role of nTregs in HIV persistence.

## Ethics Statement

This study was carried out in accordance with the recommendations of Declaration of Helsinki, Beijing 302 Hospital Research Ethnics Committee. The protocol was approved by the Beijing 302 Hospital Research Ethnics Committee.

## Author Contributions

J-WS, H-HH, CZ, F-SW, and Y-MJ conceived the study, designed the experiments, and analyzed the data. J-WS, CZ, and H-GY performed the experiments. H-HH contributed to reagents and materials. J-WS, CZ, J-YZ, R-NX, LJ, MS, F-SW, and Y-MJ wrote the article. All authors read and approved the final manuscript.

### Conflict of Interest

The authors declare that the research was conducted in the absence of any commercial or financial relationships that could be construed as a potential conflict of interest.

## Supplementary Material

Supplementary Figure 1 The expression of Foxp3 on nTreg and mTreg cells were analyzed by flow cytometry.

Supplementary Figure 2 The relationship between CD39^+^ nTregs and CD39^+^ mTregs with CD4^+^ T cell counts and viral load. Correlations of the frequency of **(A)** CD39^+^ nTregs and **(B)** CD39^+^ mTregs with CD4^+^ T cell counts. The correlations of the absolute number of **(C)** CD39^+^ nTregs and **(D)** CD39^+^ mTregs with CD4^+^ T cell counts. Correlations of the frequencies of **(E)** CD39^+^ nTregs and **(F)** CD39^+^ mTregs with viral load.
