# Supplementary figures and images for: Expression of CD39 Is Correlated With HIV DNA Levels in Naïve Tregs in Chronically Infected ART Naïve Patients
Source: Front Immunol. 2019 Oct 17;10:2465. doi: 10.3389/fimmu.2019.02465 (PMC6811520; doi:10.3389/fimmu.2019.02465)

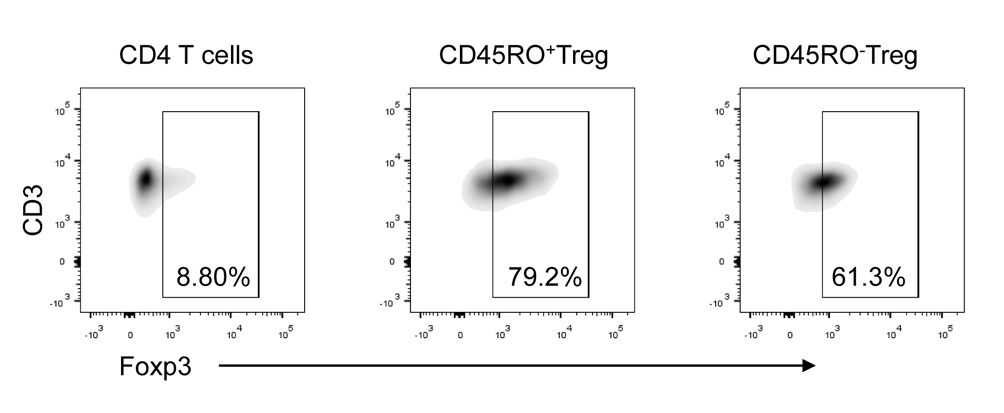

Supplement: Supplementary Figure 1 — The expression of Foxp3 on nTreg and mTreg cells were analyzed by flow cytometry. [file Image_1.TIF]

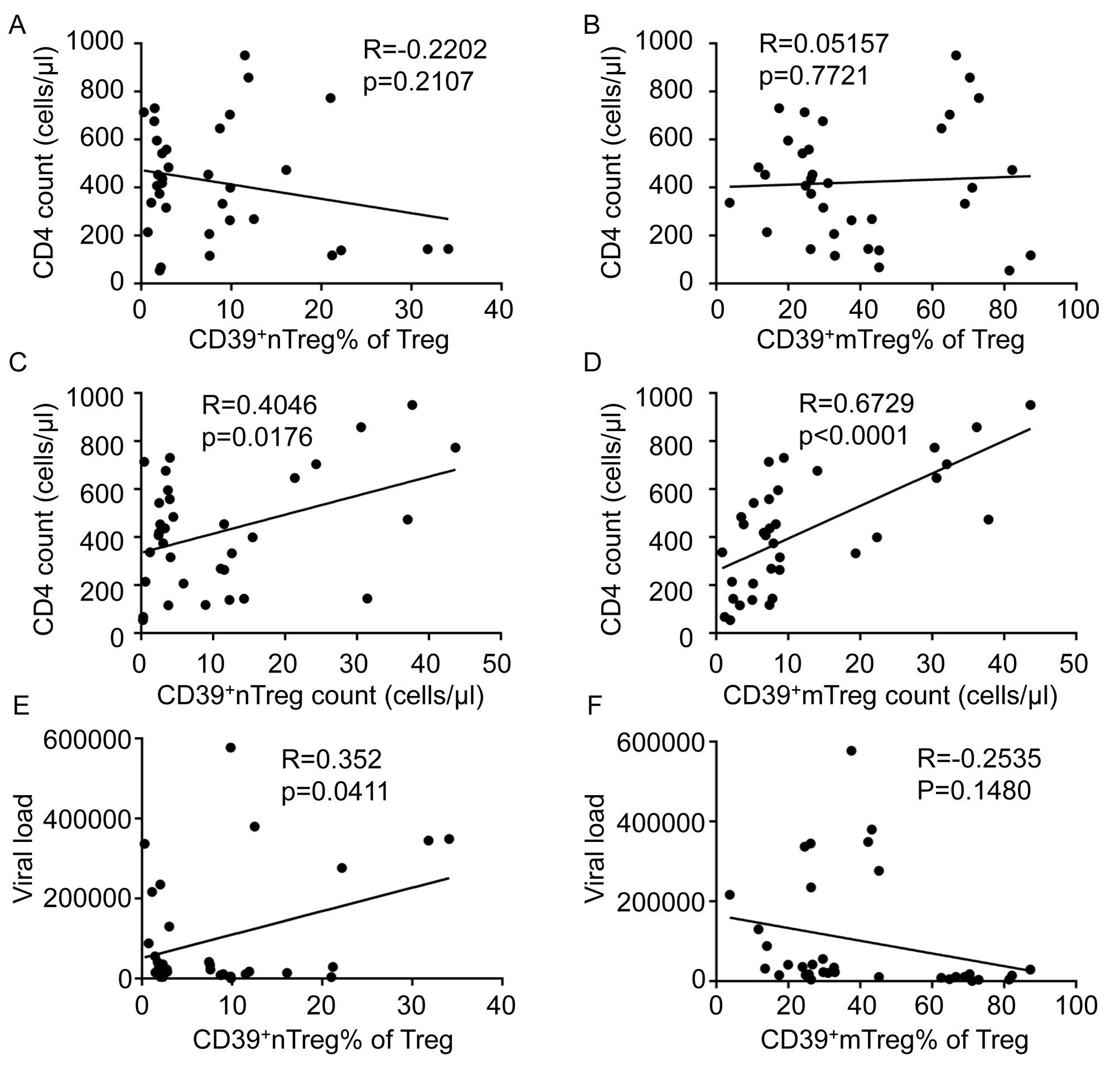

Supplement: Supplementary Figure 2 — The relationship between CD39+ nTregs and CD39+ mTregs with CD4+ T cell counts and viral load. Correlations of the frequency of (A) CD39+ nTregs and (B) CD39+ mTregs with CD4+ T cell counts. The correlations of the absolute number of (C) CD39+ nTregs and (D) CD39+ mTregs with CD4+ T cell counts. Correlations of the frequencies of (E) CD39+ nTregs and (F) CD39+ mTregs with viral load. [file Image_2.TIF]
